# Individual, High-Precision 3D Mandibular Model for Finite Element Analysis of Three-Unit Bridges: A Biomechanical Pilot Study

**DOI:** 10.3390/jfb17060285

**Published:** 2026-06-08

**Authors:** István Pelsőczi-Kovács, Bálint Deák, Klaudia Papp, Attila István Piros

**Affiliations:** 1 Department of Prosthodontics, Faculty of Dentistry, University of Szeged, Tisza Lajos körút 64–66, H-6720 Szeged, Hungary; deak.balint@szte.hu; 2 Department of Innovative Vehicles and Materials, GAMF Faculty of Engineering and Computer Science, John von Neumann University, Izsáki út 10., H-6000 Kecskemét, Hungary; papp.klaudia@nje.hu; 3 Department of Machine and Product Design, Faculty of Mechanical Engineering, Budapest University of Technology and Economics, Műegyetem rkp. 3., H-1111 Budapest, Hungary

**Keywords:** CAD image reconstruction, fixed partial denture, finite element analysis (FEA), NURBS, patient-specific modelling, periodontal ligament, mandibular biomechanics, CBCT, dental prosthesis design, geometric simplification, restorative dentistry

## Abstract

Tooth-supported fixed partial dentures (FPDs) exhibit complex biomechanical behaviour because occlusal loads are transferred through the periodontal ligament (PDL) and heterogeneous mandibular bone. This pilot study aimed to develop a patient-specific NURBS-based finite element analysis (FEA) workflow for anatomically realistic mandibular reconstruction and to evaluate the biomechanical effect of geometric simplification in tooth-supported FPD simulations. Cone beam computed tomography data from a single subject were segmented and reconstructed into a layered three-dimensional model of the mandible and dentition, including cortical bone, cancellous bone, teeth, and PDL. A high-fidelity reference model (V0) and four simplified variants (V1–V4) were analysed under static 500 N loads applied at 0° and 30°. The reference model yielded a maximum von Mises stress of 507 MPa and a peak displacement of 0.74 mm, with stress concentrations consistently localised at the retainer–pontic connector region. Inclusion of the PDL markedly affected the mechanical response, doubling denture displacement in simplified comparative models. Among the simplified configurations, V4, which preserved cortical morphology and PDL representation while omitting detailed trabecular architecture, showed the closest agreement with the reference model, with mean deviations of 6.1% and 5.8% under the two loading conditions, respectively. These findings suggest that patient-specific NURBS–FEA modelling provides a robust framework for biomechanical assessment of tooth-supported FPDs, while controlled simplification may improve computational efficiency without substantially compromising accuracy under static loading conditions.

## 1. Introduction

### 1.1. Clinical Background

The long-term clinical success of fixed partial dentures depends on the complex interaction among prosthetic design, the supporting tooth structures, and the surrounding biological environment [1,2]. Predicting the mechanical behaviour of these restorations within the oral cavity remains challenging, particularly for conventional tooth-supported restorations, in which occlusal loads are transmitted through natural abutment teeth rather than through rigid osseointegrated implants [3]. Unlike osseointegrated implants, which are rigidly anchored to bone, natural teeth exhibit physiological mobility because of the presence of the periodontal ligament (PDL), resulting in non-uniform load transfer and time-dependent stress distribution within both the prosthetic structure and the supporting hard tissues [4,5]. Accordingly, tooth-supported restorations demonstrate fundamentally different biomechanical behaviour under functional loading [3]. Clinical complications such as loss of retention, abutment tooth overload, secondary caries, and periodontal deterioration are frequently associated with unfavourable stress concentrations in tooth-supported restorations [6]. These biomechanical responses are strongly influenced by abutment tooth characteristics, including periodontal status and alveolar bone conditions. However, from a biomechanical perspective, their influence on stress distribution under occlusal loading remains insufficiently understood.

### 1.2. Scientific Background of Digital Modelling

Medical imaging techniques, such as computed tomography (CT), provide highly detailed visualisations of the human mandible and dental structures [7]. At the same time, CT data serve as a fundamental basis for the reconstruction of complex anatomical structures [8]. Among these reconstruction tasks, the mandible represents a particular challenge due to its intricate geometry and the necessity to distinguish between cortical layer and cancellous bone structure. Accurate modelling of these segments is essential for reliable biomechanical simulations.

Early three-dimensional reconstruction methods offered limited accuracy and relied largely on manual morphometric spline modelling for personalised skeletal reconstructions, particularly in the spine [9]. Subsequently, more advanced approaches improved automation through filtering-based volume segmentation using three-dimensional multi-resolution wavelets [10] and deep learning-based methods for downsampled CT geometric modelling, such as DsigNet [11]. Iterative algorithms, including MLEM [12], have further improved reconstruction quality and PET convergence through voxel-based geometries. Currently specialised software is widely used to generate three-dimensional geometric models from two-dimensional DICOM data, including methods based on empirical point-cloud acquisition and patient-specific periodontal finite element mesh generation [13,14]. Conformal geometric algebra (CGA) represents a promising future direction for multi-vector-based medical imaging [15].

Overall, early approaches prioritised manual precision, whereas more recent methods favour the scalability of artificial intelligence. However, deep-learning feature-based similarity metrics often generalise poorly across imaging modalities and may be computationally inefficient. For biomechanical applications, hybrid strategies may therefore offer the most effective compromise between personalisation and computational efficiency, although such approaches have not yet been fully incorporated into currently available reconstruction software.

**Figure 1:**
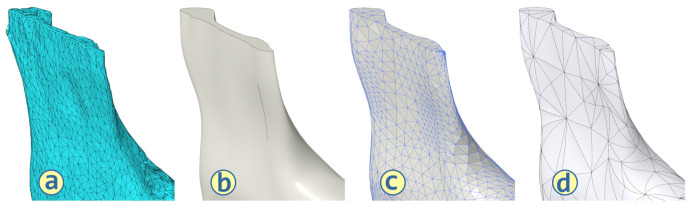
Relationship of base geometry and FEA mesh quality, (**a**) original tessellated geometry, (**b**) reconstructed NURBS geometry, (**c**) mesh with polygonal elements, (**d**) mesh with geometric elements.

Mathematical splines are widely used for the reconstruction of biological structures, such as arterial networks [16]. Among these, Bézier curves [17] and Non-Uniform Rational B-Splines (NURBS) [18] have facilitated the integration of modern computer-aided design (CAD) systems into biomedical modelling. These parametric modelling methods have become indispensable tools for high-precision geometric reconstruction. NURBS curves and surfaces enable smooth and accurate representations of anatomical forms, while allowing incorporation of complex morphological features and internal structures. Compared to conventional tessellated models, which often suffer from limited flexibility and reduced accuracy, NURBS-based reconstructions offer superior adaptability. They also support the generation of high-quality volumetric meshes for advanced simulations and computational analyses. In this context, conventional finite element analysis may be limited by its dependence on discretised anatomical geometry. By contrast, the incorporation of curved elements and NURBS-based geometric representations preserve geometric continuity, reduces the need for excessive mesh refinement (Figure 1), and mitigates errors associated with geometric approximation [19].

An accurately reconstructed three-dimensional (3D) geometric model can serve as the basis for rapid computer numerical control (CNC) tooling, as well as for partial [20] or full [21] finite element analysis (FEA). Within FEA, a well-defined CAD model allows distinct material properties to be assigned to individual structural layers. Such segmentation enables realistic simulation studies aimed at evaluating the mechanical response of the mandible under various loading conditions.

### 1.3. Knowledge Gap

Finite element (FE) analysis has become a widely accepted tool for investigating dental biomechanics; however, the majority of published FE studies have focused on implant-supported fixed [22,23,24,25,26] or removable [25] restorations. Finite element analysis of fixed prostheses typically uses detailed three-dimensional models to evaluate fracture risk and load distribution, highlighting the critical role of geometric accuracy in obtaining reliable mechanical simulations. One reason for this predominance may be the relative simplicity of implant modelling, as implants are commonly assumed to be ankylotically fixed within the surrounding bone. In contrast, the biomechanical modelling of natural teeth is considerably more complex because it requires accurately representation of the periodontal ligament as well as the heterogeneous structure of the surrounding hard tissues [26]. Only a limited number of FE studies have addressed conventional tooth-supported fixed partial dentures, and even fewer have incorporated high-accuracy, patient-specific geometries that faithfully represent the layered mandibular structure and the anatomical integration of teeth within bone [27].

Eric et al. (2024) [22] used simplified finite element models with standardised prosthetic geometries to assess stress distribution in fixed partial dentures across different materials and support types, but without patient-specific anatomical detail. Koosha and Mirhashemi (2013) [23] advanced this approach by modelling three distinct tooth-implant-supported fixed prosthesis designs in idealised mandibular sections with homogeneous bone properties, thereby enabling parametric comparisons using three-dimensional FE analysis. Ziada and Beleidy (2025) [24] incorporated customised abutment geometries with varying internal implant–abutment connections and simulated stress distributions in simplified peri-implant environments to evaluate material-related effects. Ceddia et al. (2025) [25] focused on ultra-short implants, employing axisymmetric FE models to capture insertion torque effects on peri-implant bone stress interfaces and also examined three-implant splinted prostheses with detailed modelling of crestal bone positioning in idealised settings.

Overall, these studies reflect a progression from generic standardised models toward more customised, interface-specific reconstructions. Nevertheless, they continue to rely largely on idealised rather than patient-derived jaw anatomies, thereby limiting clinical translation in the presence of real-world morphological variability. Moreover, the transfer of stress to the prosthesis, abutment teeth, and surrounding bone has not yet been systematically evaluated using anatomically detailed three-dimensional models. Some recent studies have increasingly adopted digital workflows, including digital impressions [28], and CAD-based techniques [3], using high-fidelity geometric models that prioritise accuracy for realistic FEA simulations of mechanical behaviour in conventional fixed restorations. Other studies have explored the integration of artificial intelligence for model optimisation or the use of micro-CT scanning to obtain highly accurate geometries [29]. Precise computational modelling of prosthesis geometry may facilitate more predictable assessment of long-term mechanical performance and longevity [30].

The primary strength of present study lies in the use of high-fidelity, patient-specific three-dimensional models that accurately represent the layered jaw structure and anatomical integration of teeth within the bone. In this way, the study addresses a notable gap in the still limited finite element literature on conventional tooth-supported partial dentures [27]. At the same time the present work should be interpreted primarily as a methodological and comparative biomechanical investigation, with model behaviour assessed against published contemporary data rather than as a fully predictive clinical model [31,32].

### 1.4. Aim of the Study

The present pilot study focuses exclusively on the development of a periradicular anchorage model, including the periodontal ligament and surrounding bone structures, for finite element analysis (FEA) and for evaluation of its biomechanical applicability. Computed tomography (CT)-based imaging enables reconstruction of high-precision three-dimensional (3D) tooth–jaw models that can be converted into geometric representations suitable for finite element modelling. A carefully reconstructed NURBS surface also provides an opportunity for customised remeshing of the geometry. In conventional automated workflows, segmented CT data are typically used directly, and the accuracy of the finite element mesh is therefore strongly dependent on the quality of the segmented three-dimensional dataset. In contrast, the present study introduces a novel reconstruction approach based on two-dimensional slices, which may offer a faster and more accessible route for geometric reconstruction of the mandible.

The study also aims to investigate the stress distribution in tooth-supported partial dentures under bidirectional static loading using finite element analysis. To this end, a patient-specific, high-precision three-dimensional mandibular model was systematically modified, in order to evaluate the biomechanical consequences of progressive model simplification. This pilot study was not designed to provide a detailed analysis of the biomechanics of the PDL-bone interface adjacent to fixed partial denture (FPD) abutments, nor to investigate occlusal contact behaviour during dynamic masticatory function. Rather, its main purpose was to examine the methodological feasibility of the proposed modelling strategy and to compare stress patterns across simplified model configurations. Experimental support for the finite element results was limited and was focused primarily on the observed stress patterns in the simplified models, while the numerical findings were also compared with values reported in the contemporary literature [31,32,33,34,35].

The long-term objective of this research programme is to translate the developed NURBS–FEA framework into a clinically applicable methodology. More specifically, this includes the use of patient-specific CBCT data to support quantitative assessment of the long-term biomechanical behaviour of planned dental prostheses, such as fixed partial dentures (FPDs) and removable partial dentures (RPDs), in relation to each individual’s biological characteristics (e.g., bone quality and periodontal ligament properties) and morphological features (e.g., mandibular geometry and trabecular architecture). Further refinement of the methodology may enable its integration into digital prosthetic design workflows. Additional opportunities for development include the analysis of stress concentration patterns and the identification of potential risk zones within digitally assisted, patient-specific treatment planning workflows.

## 2. Materials and Methods

### 2.1. CT Segmentation

Segmentation of cone beam computed tomography (CBCT) data constitutes a critical preprocessing step in the digital reconstruction of mandibular and dental anatomy. The study was conducted in accordance with the Declaration of Helsinki and was based on data from CBCT scans of a single patient (one of the authors) taken at the clinic between 1 January 2020 and 31 March 2026. Given the local regulatory framework, no separate ethical approval (No. 82/SZTE RKEB/2026) was required for the retrospective data used in this pilot study. The objective of the segmentation procedure was to accurately delineate individual anatomical components, including the mandible, tooth roots, and internal bony structures, in order to generate geometries suitable for biomechanical modelling and finite element analysis (FEA). Segmentation was performed using the 3D Slicer software platform (version 5.6.2; https://www.slicer.org). The reconstructed CT dataset was evaluated in three orthogonal planes—sagittal, axial, and coronal—to ensure geometric consistency and anatomical accuracy. These views enabled continuous verification of segmented boundaries and facilitated comparison between two-dimensional slices and the evolving three-dimensional reconstruction.

**Figure 2:**
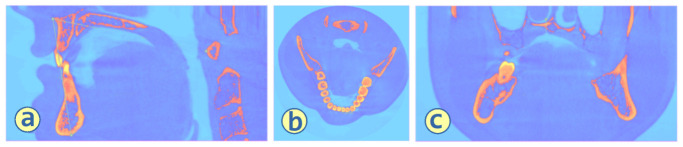
Sagittal (**a**), axial (**b**), and coronal (**c**) section of the mandible.

Sagittal slices provided information on the longitudinal arrangement of mandibular and dental structures (Figure 2a). Axial sections enabled detailed inspection of internal features such as the mandibular canal and trabecular bone morphology (Figure 2b). Coronal views supported assessment of cortical bone thickness and the spatial relationship between teeth and surrounding bone tissue (Figure 2c).

The segmentation process resulted in a layered three-dimensional model in which the mandible and dental structures were represented as separate anatomical regions. This representation allows the assignment of distinct material properties to individual components and serves as the geometric basis for subsequent finite element simulations. The generated model provides a consistent and anatomically realistic framework for further numerical and biomechanical investigations.

### 2.2. Parametric 3D Reconstruction of the Mandible

Parametric three-dimensional reconstruction of the mandible was performed to obtain a geometrically accurate model suitable for biomechanical analysis and finite element simulations. The reconstruction process was based on segmented CT datasets in which the principal anatomical components, including cortical, and trabecular bone regions, were clearly identified.

**Figure 3:**
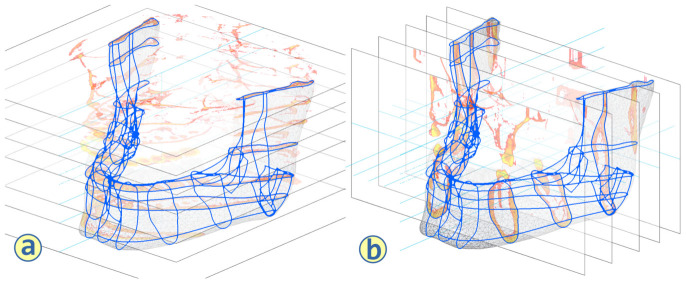
Fitting axial (**a**) and cortical (**b**) CT sections on the mandible described by curves.

The reconstruction was performed using PTC Creo 12.4 (PTC Inc., Boston, MA, USA), selected for its advanced parametric and NURBS-based surface modelling capabilities. Cross-sectional CT images served as geometric references for defining interpolation curves representing the mandibular contours. NURBS curves were generated from the segmented boundaries by fitting control points to the extracted anatomical profiles, thereby ensuring close correspondence between the reconstructed geometry and the original CT data (Figure 3).

These NURBS curves were subsequently interconnected to form a continuous network of quadrilateral surface patches. This surface-modelling strategy ensured smooth transitions between adjacent regions and eliminated geometric discontinuities. The resulting surface representation was then converted into a solid model, providing a consistent volumetric geometry suitable for numerical analysis and mesh generation.

Particular emphasis was placed on preserving the parametric nature of the model, thereby allowing systematic modification of geometric features for subsequent computational studies and sensitivity analyses. This flexibility is essential for evaluating different loading conditions, prosthetic configurations, and design variations within a unified modelling framework.

**Figure 4:**
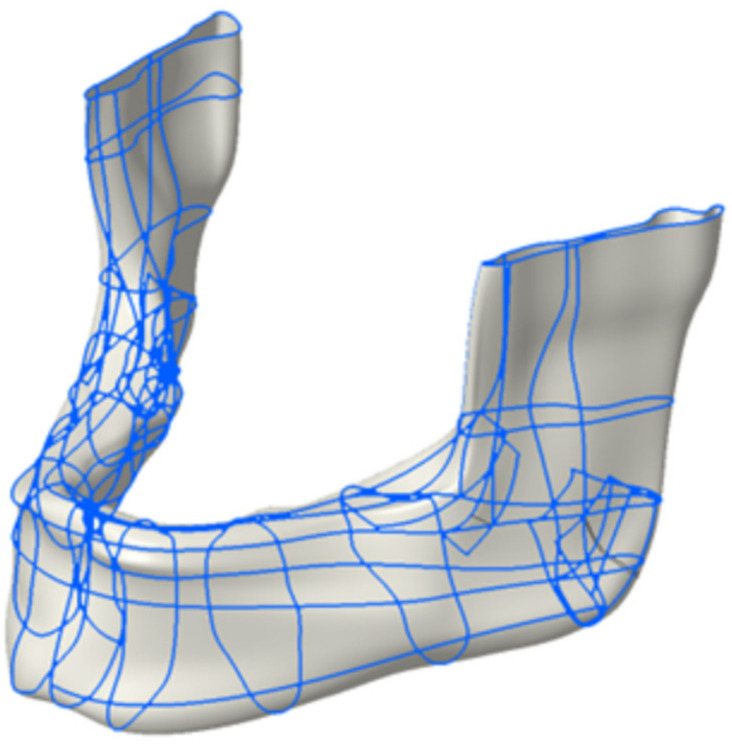
Fit of the mandible described by NURBS curves on the final 3D model.

The layered anatomical structure of the mandible was explicitly represented by separating cortical and trabecular bone regions within the model. This approach enables the assignment of distinct material properties to each anatomical component and improves the physiological relevance of the simulated mechanical response. The finalised model accurately reproduces both the external cortical surface and the internal geometry of the mandible (Figure 4).

**Figure 5:**
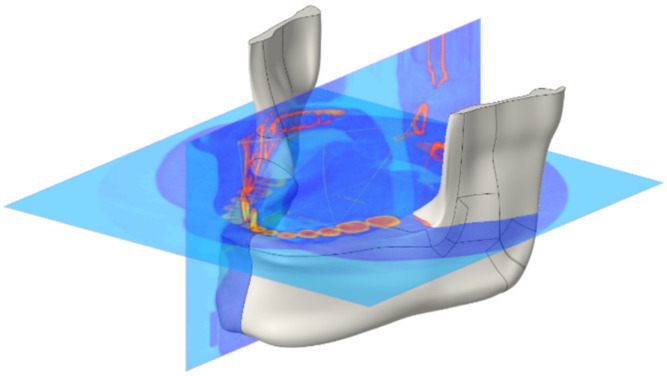
Combined visualisation of the processed data.

The NURBS-based reconstruction approach proved effective in capturing the complex mandibular morphology while maintaining a smooth and topologically consistent surface structure. Figure 5 illustrates a composite comparison of the sagittal, coronal, and axial CT slices with the corresponding reconstructed model.

The resulting parametric 3D model provides a robust geometric basis for subsequent finite element simulations and biomechanical investigations, supporting high anatomical fidelity and numerical stability in further analyses.

### 2.3. Layered Segmentation of the Mandible

The mandible exhibits a heterogeneous internal structure consisting primarily of an outer cortical layer and an inner trabecular core, which differ significantly in mechanical behaviour. Accurate representation of this layered architecture is essential for realistic biomechanical modelling and finite element simulations. Layered segmentation was performed on the previously reconstructed three-dimensional mandibular model to explicitly distinguish between cortical and trabecular bone regions. The interfaces between these layers were identified based on analysis of the CT dataset, enabling anatomically consistent separation of the two structural components. This subdivision allows the assignment of distinct material properties to each region in subsequent numerical analyses.

**Figure 6:**
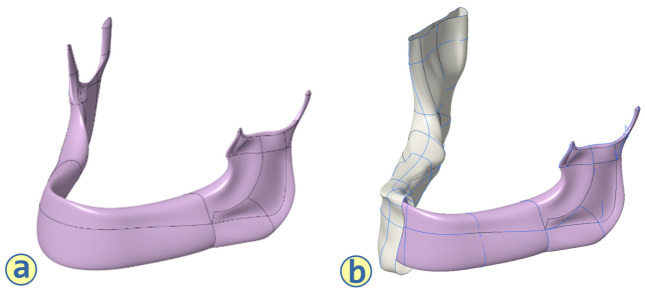
The inner trabecular (cancellous) region of the jaw (**a**) and its anatomical relationship to the overlying compact cortical layer (**b**).

The trabecular bone layer was generated by applying a geometric offset to the external cortical surface. This procedure ensured that the internal part followed the overall morphology of the mandible while maintaining geometric continuity and avoiding topological inconsistencies. Careful control of the offset distance allowed preservation of anatomical proportions and ensured compatibility with finite element mesh generation. Figure 6a illustrates the reconstructed trabecular region derived from the CT data, while Figure 6b shows its spatial relationship within the compact cortical layer.

**Figure 7:**
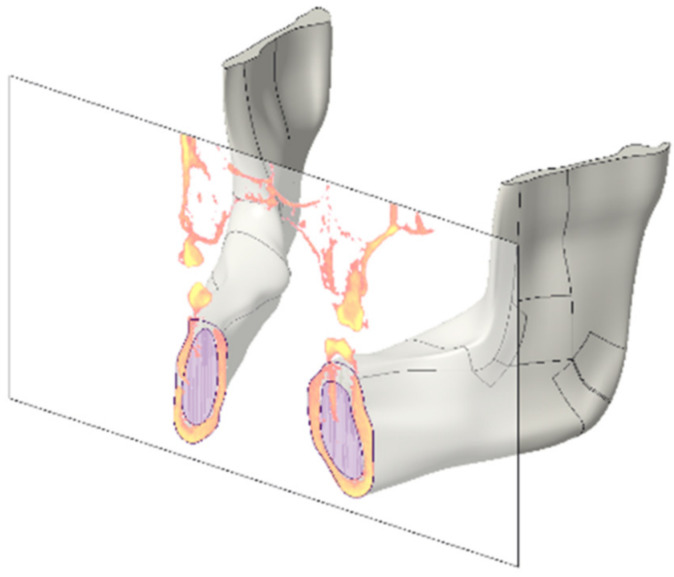
The geometry with CAD data placed on the coronal section.

The reconstructed geometry closely reflects the anatomical structure captured by the CT scans. The original voxel-based imaging data, representing the spatial distribution of tissue density, were converted into a surface-based representation through surface reconstruction techniques. This procedure preserved the anatomical fidelity of the imaging data while yielding a geometry suitable for computer-aided design (CAD) operations and numerical modelling. Figure 7 illustrates the correspondence between a segmented coronal CT slice and the respective region of the reconstructed digital mandible, thereby confirming the geometric consistency of the final model with the underlying imaging data.

The CAD model captures the spatial relationships among the cortical shell, trabecular core, and dental structures by means of NURBS-based curves and surfaces, thereby preserving smooth geometric transitions and ensuring the accurate alignment of anatomical features.

### 2.4. Parametric 3D Reconstruction of the Tooth

The geometric reconstruction of individual teeth followed a workflow analogous to that used for the mandible; however, a higher level of geometric detail and manual refinement was required due to the complex morphology of dental structures. CT-based tooth geometries provide patient-specific anatomical information but typically contain surface irregularities such as artificial cavities, sharp edges, and local distortions. These artifacts necessitated systematic geometric correction to ensure smooth and anatomically consistent surfaces suitable for numerical analysis.

**Figure 8:**
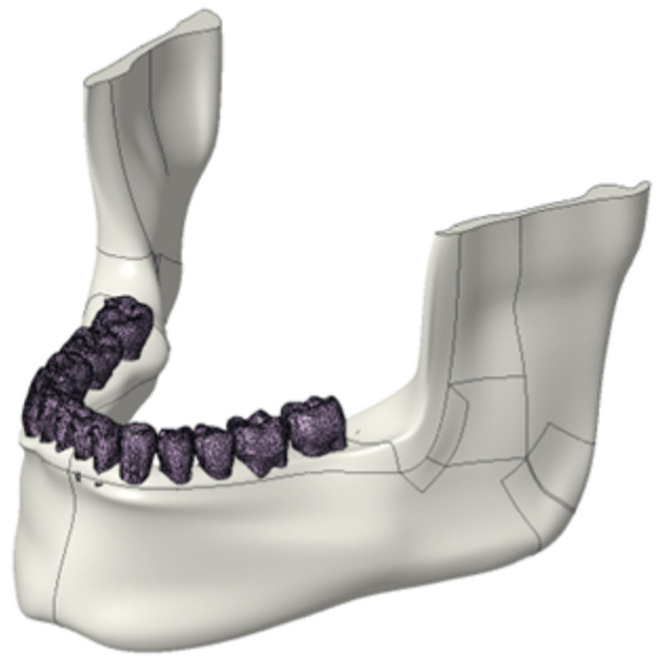
Position of the integrated teeth within the mandible.

As an initial step, the tooth geometries extracted from the CT dataset were imported into the CAD environment and positioned within the previously reconstructed mandible model. This integration served as a spatial reference system, ensuring anatomically correct placement of each tooth within the alveolar ridge (Figure 8).

For geometric reconstruction, each tooth was analysed in three orthogonal sectional planes: axial, sagittal, and coronal. Multiple cross-sectional slices were generated in each view to capture the overall morphology and local geometric variations. NURBS-based interpolation curves were manually defined along the segmented contours derived from the CT images, providing an accurate representation of the tooth boundaries.

**Figure 9:**
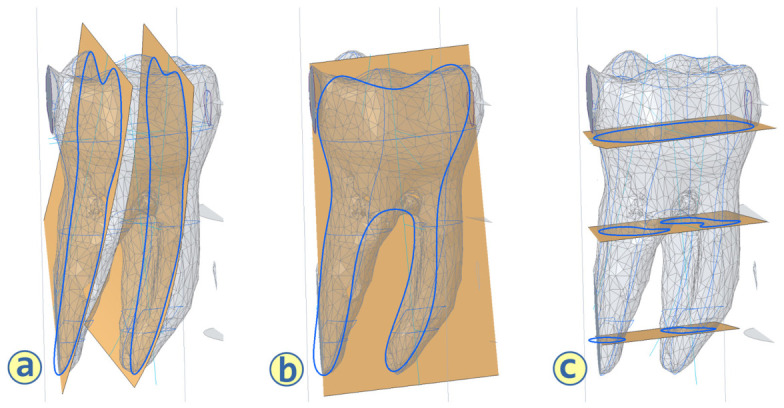
Sections and curves from the cortical (**a**), sagittal (**b**), and axial (**c**) view.

Particular attention was devoted to correcting scanning artifacts and irregular contour features. Local distortions, such as false cavities or exaggerated protrusions, were replaced with smooth, anatomically realistic profiles. To ensure surface continuity, each closed contour was constructed from at least two tangentially connected curve segments rather than a single curve. This strategy improved geometric smoothness and reduced the risk of discontinuities during surface generation (Figure 9a). In the coronal plane, two sectional curves were defined for multi-rooted teeth to enable separate reconstruction of the individual roots (Figure 9b). In contrast, a single sectional curve was considered sufficient in the sagittal plane, as tooth geometry exhibits limited variation along this direction (Figure 9c). In the axial plane, three sections were generated to capture geometric changes along the tooth axis: one before root bifurcation, one immediately after separation, and one near the apical region of the roots.

**Figure 10:**
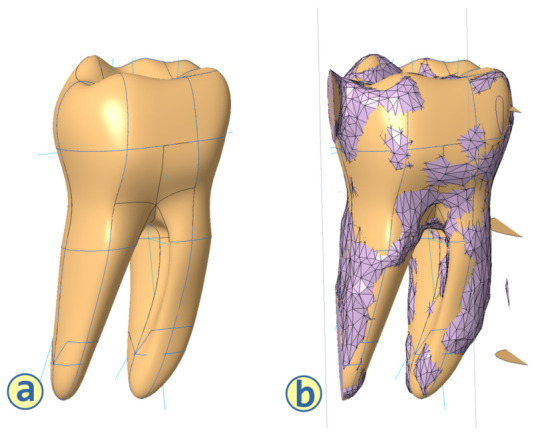
3D model of the tooth (**a**) and the comparison of the resulting tooth model with the CT-based model (**b**).

Following contour definition, surfaces were generated between corresponding curves using quadrilateral NURBS surface patches, consistent with the methodology applied for mandibular reconstruction. This approach ensured smooth transitions between adjacent sections and resulted in a topologically consistent surface model. The assembled surfaces formed a closed, anatomically realistic three-dimensional tooth geometry suitable for solid modelling and finite element mesh generation (Figure 10a). To evaluate reconstruction accuracy, the remodelled tooth geometry was compared with the corresponding tooth surface derived directly from the CT dataset. This comparison enabled both visual and quantitative assessment of geometric fidelity and dimensional consistency (Figure 10b).

**Figure 11:**
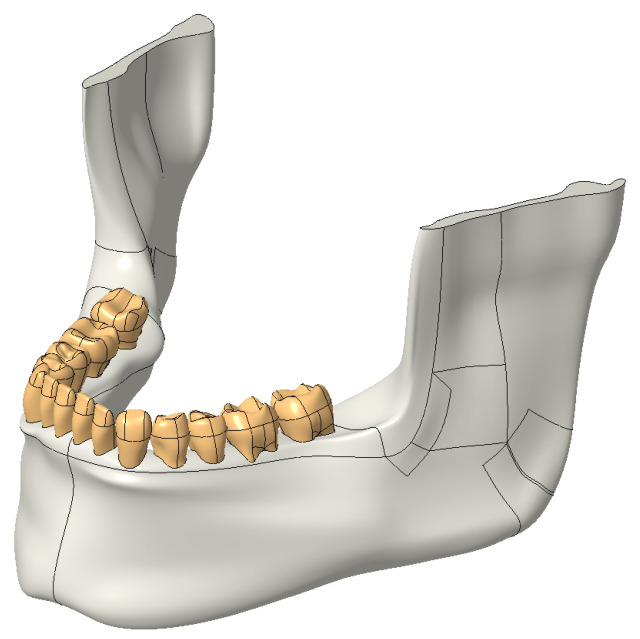
Teeth remodelled in the mandible.

Subsequently, all mandibular teeth were reconstructed using the same procedure and integrated into the mandibular model. The complete dentition embedded within the reconstructed mandible is shown in Figure 11, illustrating consistent anatomical alignment and geometric uniformity across all reconstructed tooth models.

### 2.5. Construction of a High-Fidelity Clinical Model with Periodontal Ligament (PDL) Reconstruction

Although many FE studies often do not explicitly include the periodontal ligament (PDL), it is now widely accepted that the PDL plays a key role in transmitting occlusal forces from the teeth to the alveolar bone. Various analytical models, approaches, and assumptions have been employed in finite element analyses of the PDL. In most studies, homogeneous linear or nonlinear models and their variants—such as isotropic or anisotropic, and elastic or poroelastic formulations—predominate in terms of material representation [33]. More recent studies have adopted hyperelastic material models with near-hydrostatic pressure behaviour [34], including hyperelastic Ogden-type formulations [35].

Only a limited number of in vivo studies have investigated the elastic properties of the human periodontal ligament. Although these studies, conducted primarily on maxillary central incisors, have highlighted the nonlinear elastic behaviour of the PDL, they were mainly designed for orthodontic applications under continuous dynamic loading [36]. In review-based comparative analysis of the PDL, linear material properties have often been selected as a practical basis for FE modelling [37]. Accordingly, in the present finite element study, the PDL was represented as a homogeneous isotropic solid layer, consistent with standard approaches reported in the literature [38]. Poroelastic PDL material assignment are used only infrequently [33,39], whereas iscoelastic material models are typically applied in time-dependent simulations such as orthodontic studies [40,41,42,43]. Since the narrow anatomical space of the PDL permits only limited deformation under the present loading condition, an appropriately calibrated linear elastic material model was considered a reasonable approximation.

**Figure 12:**
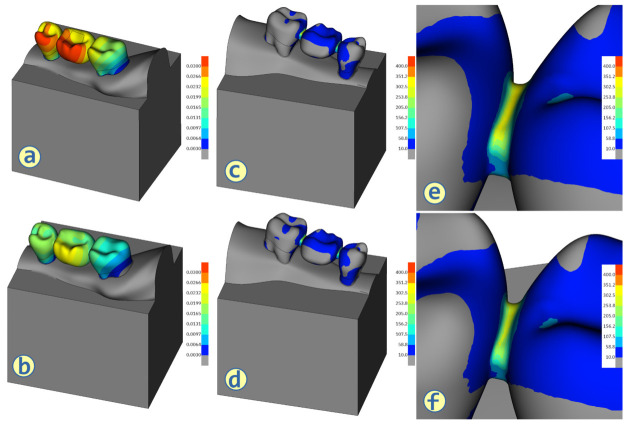
Validation study in SIMULIA Abaqus FE system, (**a**) maximum linear elastic displacement 0.036 mm, (**b**) maximum hyperelastic displacement 0.025 mm, (**c**,**e**) maximum linear von Mises stress 441.1 MPa, (**d**,**f**) maximum hyperelastic von Mises stress 431.5 MPa, linear elastic material properties: E = 66.7 MPa, ν = 0.49, hyperelastic material properties (Ogden model): μ_1_ = 160 MPa, μ_2_ = 40 MPa, α_1_ = 2.0, α_2_ = −2.0, D_1_ = 0.001 (l/MPa), D_2_ = 0.001 (l/MPa).

To support this assumption the present study included a comparison between hyper elastic and linear elastic PDL material models using Dassault Systemes SIMULIA Abaqus 2020 software (Dassault Systèmes, Vélizy-Villacoublay, France). The results indicated that, under the static loading conditions investigated, the linear elastic material model provided acceptable agreement with the hyper elastic formulation (Figure 12).

**Figure 13:**
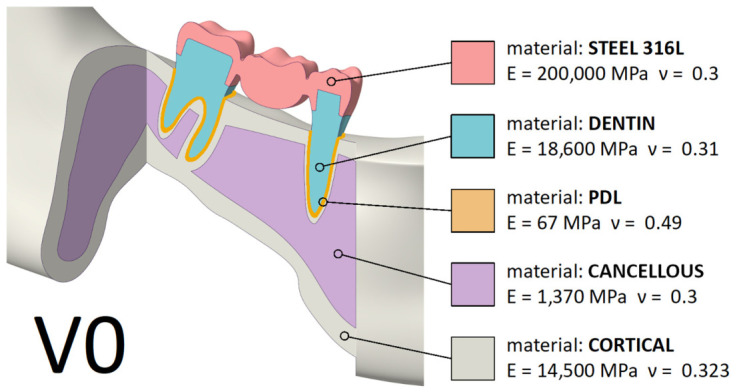
Detailed version (V0) of human mandible with fixed restoration, the colour-coded materials display the different properties of the simulation components: STEEL 316L (E = 200,000 MPa, ν = 0.3) DENTIN (E = 18,600 MPa, ν = 0.31), PDL (E = 66.7 MPa, ν = 0.49), CANCELLOUS (E = 1370 MPa, ν = 0.3), CORTICAL (E = 14,500 MPa, ν = 0.323).

In this study, a high-fidelity, patient-specific mandibular model (V0) was first generated (Figure 13), preserving the original anatomical geometry and explicitly representing cortical and trabecular bone, including the alveolar cortical bone (lamina dura), the prepared abutment teeth, and the PDL. This model (Figure 13) served as a biomechanically realistic basis for subsequent FEA of the fixed partial denture system.

**Figure 14:**
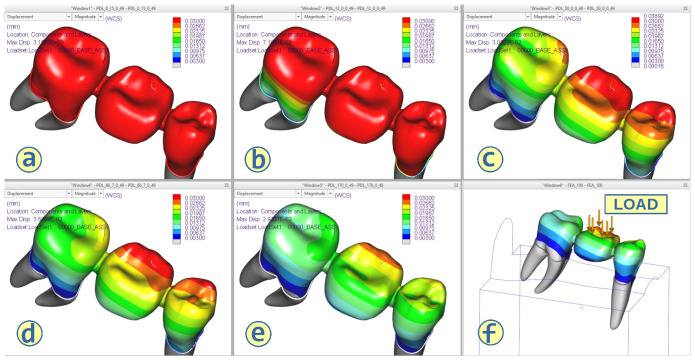
Maximum displacement values of sensitivity study in PTC Creo Simulate FE system, (**a**) 3.157 mm/E = 0.15 MPa, (**b**) 0.071 mm/E = 12 MPa, (**c**) 0.039 mm/E = 50 MPa, (**d**) 0.036 mm/E = 66.7 MPa, (**e**) 0.029 mm/E = 175 MPa, (**f**) simulation arrangement with load.

**Figure 15:**
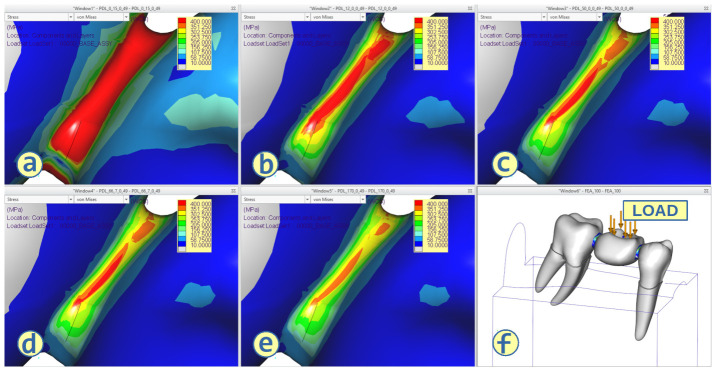
Maximum von Mises stress values of sensitivity study in PTC Creo Simulate FE system, (**a**) 2835 MPa/E = 0.15 MPa, (**b**) 573.3 MPa/E = 12 MPa, (**c**) 512.8 MPa/E = 50 MPa, (**d**) 503.1 MPa/E = 66.7 MPa, (**e**) 494.9 MPa/E = 175 MPa, (**f**) simulation arrangement with load.

The PDL geometry was reconstructed using a thin offset of the root surfaces, with an average offset distance of 0.2 mm. During preprocessing for numerical analysis, specific material properties of E = 66.7 MPa, ν = 0.49 were assigned to this layer. The relevant literature reports a broad range of elastic moduli for the PDL, approximately 0.15–175 MPa, and Poisson’s ratios ranging from 0.30 to 0.49 [37,44,45]. To evaluate the influence of these values on the PTC Creo FE model, a sensitivity analysis was performed. This analysis supported the selected elastic modulus value of 66.7 MPa within a broad range of linear elastic material properties. The results showed stable convergence of the mechanical response, with deviations ranging from approximately −1.6% to 1.9% between 50 and 175 MPa (Figure 14 and Figure 15).

### 2.6. Construction of Simplified Clinical Models

Although the detailed V0 model offers a high degree of anatomical fidelity, its computational complexity, arising from the intricate geometries and topologies extracted from high-resolution CT scans, presents challenges for rapid prototyping and parametric studies. To address this issue, a series of progressively simplified models (V1–V4) was developed, enabling faster mesh generation, reduced simulation times, and broader applicability in clinical research and design optimisation. These variants allow for rigorous quantitative comparisons between the gold standard “physiological simulator” (V0) and the simplified archetypes commonly encountered in the previous finite element analysis literature, thereby offering a simpler protocol while maintaining accuracy and efficiency.

**Figure 16:**
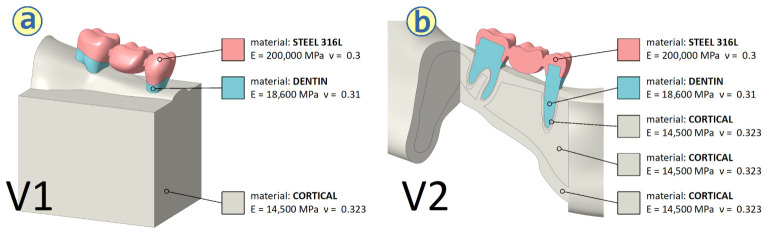
The teeth are placed in a solid block; (**a**) V1 and in the form of a jawbone (**b**) V2, each consist of cortical bone.

**Figure 17:**
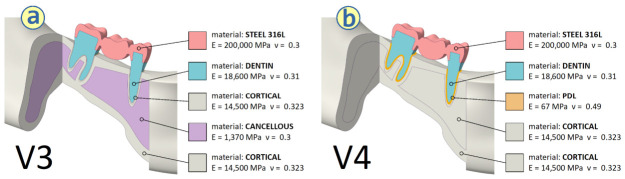
The reconstructed V3 jaw geometry with a cancellous core without PDL (**a**) and V4 structure with compact bone and PDL (**b**).

- V1 model: represents the most rudimentary approximation, in which the abutment teeth are embedded within a monolithic solid block assigned material properties equivalent to those of cortical bone (e.g., Young’s modulus of approximately 15–20 GPa). All five outer faces of the block are fully constrained by zero-displacement boundary conditions, thereby mimicking a rigidly fixed support. Although this configuration is computationally simple, it neglects the heterogeneity of the jawbone. (Figure 16a).
- V2 model: introduces basic jaw morphology by placing the teeth within a reconstructed solid volume corresponding exclusively to the cortical bone envelope (Figure 16b), without internal trabeculation. This approach enhances geometric realism while maintaining model simplicity.
- V3 model: further refines the representation by incorporating a spongy core within the reconstructed jaw geometry to simulate cancellous (trabecular) bone properties, such as a lower elastic modulus (approximately 1–2 GPa) and higher porosity. The teeth are embedded accordingly, thereby providing a closer approximation of the bone density gradients observed in vivo. (Figure 17a).
- V4 model: incorporates the teeth into a complete solid mandibular geometry (Figure 17b) while explicitly including a PDL layer reconstructed as in V0. This hybrid configuration offers a more physiologically realistic representation of tooth–bone interactions, capturing PDL-mediated load transfer without the full trabecular complexity of V0.

Collectively, these models span a spectrum from extreme simplification (V1) to near-physiological fidelity (V4), allowing for sensitivity analyses on how geometric and material simplifications influence predicted biomechanical outcomes, such as peak stresses at the bone–implant interface or abutment tooth mobility.

## 3. Results

### 3.1. Finite Element Analysis of Fixed Partial Denture Loading

**Figure 18:**
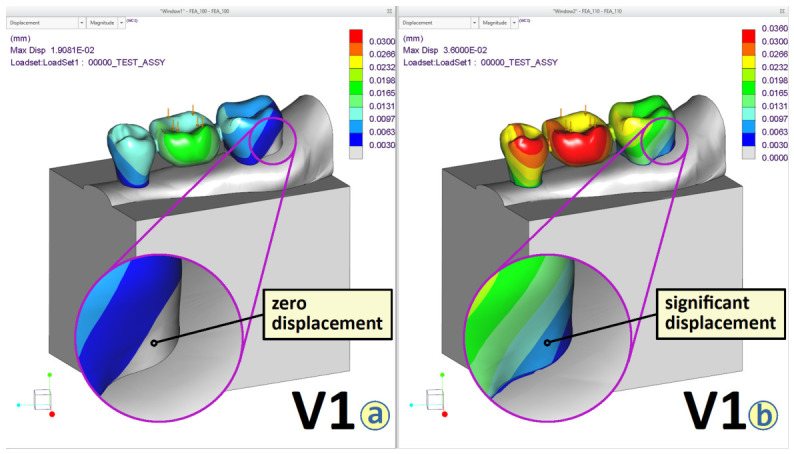
Denture displacement with highlighted tooth-neck area: PDL-excluded (**a**) vs. PDL-included (**b**) V1 FEA models.

The selected linear elastic PDL material model with quasi-incompressible formulation E = 67 MPa, ν = 0.49 provided a reasonable approximation of physiological behaviour while maintaining relatively low computational demand. The effect of the PDL layer was significant in final results, as demonstrated by comparison of models V1a and V1b. As shown in Figure 18, inclusion of the PDL created a more flexible connection between the teeth and mandible, increasing a significant denture displacement from 0.018 mm to 0.036 mm in model V1b. These findings underscore the critical role of the periodontal ligament (PDL) in transmitting occlusal forces from teeth to surrounding tissues, as evidenced by the finite element analysis [38].

Following the construction of the anatomical reference configuration (V0), four simplified models (V1–V4) were developed to evaluate how varying geometric complexity influences the biomechanical behaviour of a three-unit fixed partial denture system under functional conditions [24]. The purpose of this analysis was not direct numerical validation, but rather methodological demonstration—specifically, to identify how progressive simplifications affect stress distribution and deformation within the prosthetic assembly.

**Figure 19:**
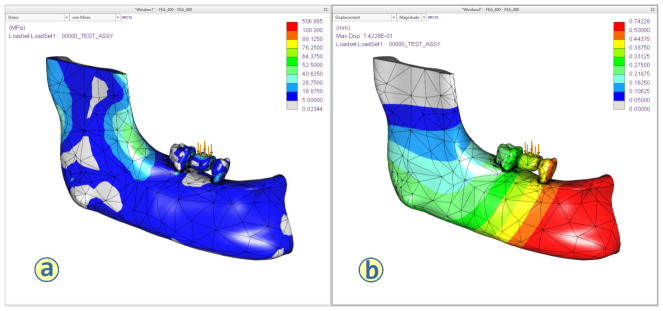
Distribution of (**a**) von Mises stress [MPa] and (**b**) displacement magnitude [mm] values of reference (V0) FEA model.

**Figure 20:**
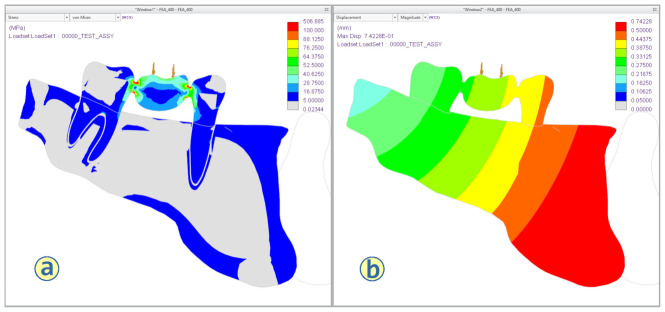
Cross sectional distribution of (**a**) von Mises stress [MPa] and (**b**) displacement magnitude [mm] values of reference (V0) FEA model.

A vertical load of 500 N was applied in the negative Y-direction, with the superior surface fully constrained, replicating physiological masticatory static loading conditions [3,4]. The detailed V0 configuration produced a maximum von Mises stress of 507 MPa and a peak deformation of 0.74 mm, localised along the mandibular midsagittal plane (Figure 19). Stress concentrations were primarily observed at the retainer-pontic connector (connector) parts (Figure 20), confirming their biomechanically critical role as stress-concentrating zones [6,30].

Cross-sectional analyses (Figure 20) revealed unloaded periodontal ligament (PDL) regions and localised stress discontinuities, reflecting realistic elastic interactions between the abutment teeth, prosthesis, and alveolar bone [4,5,27].

In the subsequent stage of the analysis, two distinct load cases were applied. The most relevant case involved a vertical load with a 0° loading angle, whereas the second case employed a 30° loading angle in accordance with ISO 14801:2016 [46].

**Figure 21:**
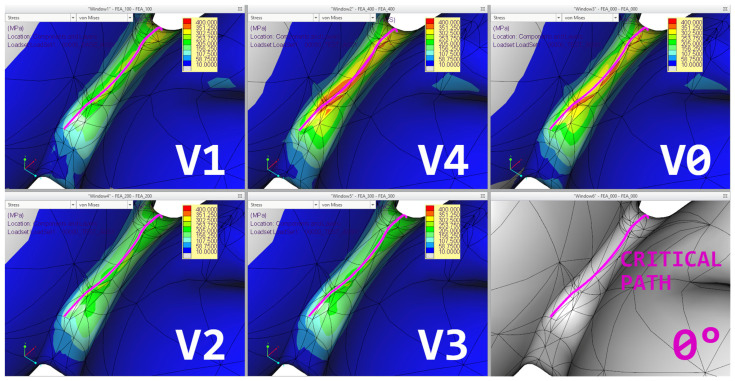
Equivalent von Mises stress distributions [MPa] around the critical bridge section with highlighted critical path (CP) in all cases (V0–V4) of FEA model at 0° loading angle.

Rather than relying on single-point result sampling, the analysis was extended to a defined region referred to as the Critical Path (CP). The CP is located at that region of denture where the critical damage was estimated based on this FE simulation. Although the evaluation of principal stress values is widely used to assess specific directional regions of the human mandible, the von Mises stress criterion is generally recommended for broader comparative analyses. The new results were presented as curves based on fifty-five sampling points, whereas only four points had been used previously. Individual values, such as deviations, were calculated using comparison of statistical average values. The application of statistical averaging reduces peak stress values; however, in short-span fixed dentures, these elevated local stresses are expected to cause only localised plastic deformation rather than permanent damage. Although the two load cases produced significantly different stress distributions, the summarised deviations showed notably similar values. In both load cases, particular attention was paid to the distribution and magnitude of stresses acting at the retainer–pontic connector interface, identified as a biomechanically critical stress-concentrating area [3,6,30], as shown in Figure 21.

**Figure 22:**
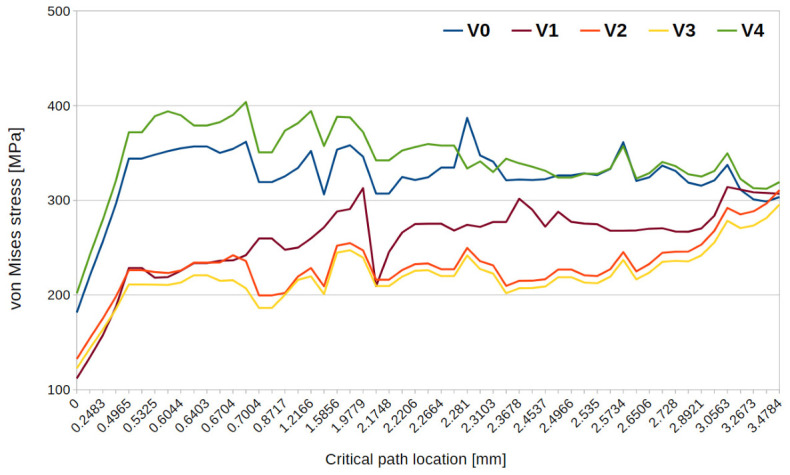
Distribution of von Mises stress values along the CP in all cases (V0–V4) of FEA model at 0° loading angle.

**Figure 23:**
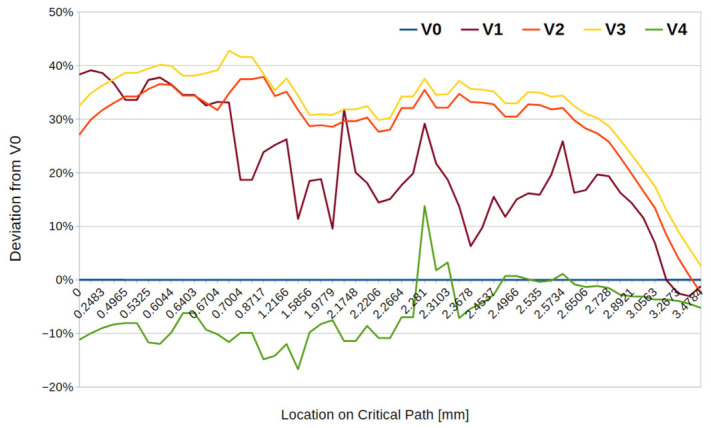
Distribution of relative differences of von Mises stress values compared to the base version (V0), the average deviation is 6.1% at V4 and 20.7%, 29.0%, 32.3% at V1, V2, V3, respectively.

Quantitative comparison of the model variants was carried out by analysing the equivalent (von Mises) stress distribution along the critical path (CP) of the prosthesis structure under a 0° loading angle (Figure 22). The relative differences shown in Figure 23, calculated with respect to the reference model (V0), demonstrated that the V4 configuration yielded the most consistent behaviour, with stress responses remaining nearly equivalent to those of the reference model and an average difference of 6.1%.

In both finite element studies, mesh convergence analysis was performed automatically using a single-pass adaptive method. In this approach, the software carried out two simulation passes and applied automatic polynomial refinement based on the initial solution. Rather than generating a large number of smaller elements, the single-pass adaptive method improves the solution by increasing the polynomial order of the geometric elements. Although both refinement approaches produce comparable results in terms of mesh quality, geometric refinement represents the original geometry more accurately. In the Single-Pass Adaptive procedure, the first pass is performed with a polynomial order of three to obtain a local estimate of the stress error. Based on this estimate, Creo Simulate determines an updated p-order distribution and then performs a final analysis. In both simulation cases, the absolute error in von Mises stress was less than 20 MPa, while the relative error in maximum principal stress remained below 3%.

**Figure 24:**
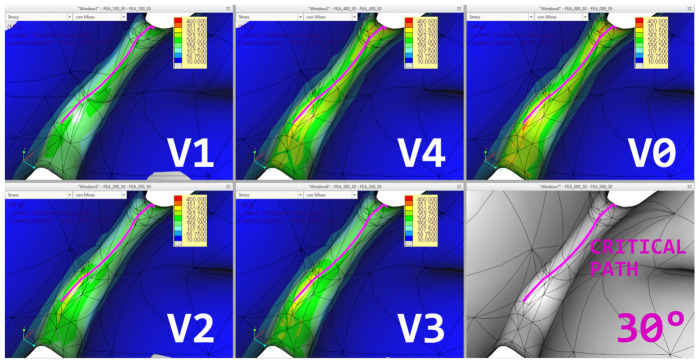
Equivalent von Mises stress distributions [MPa] around the critical bridge section with highlighted critical path (CP) in all cases (V0–V4) of FEA model at 30° loading angle.

**Figure 25:**
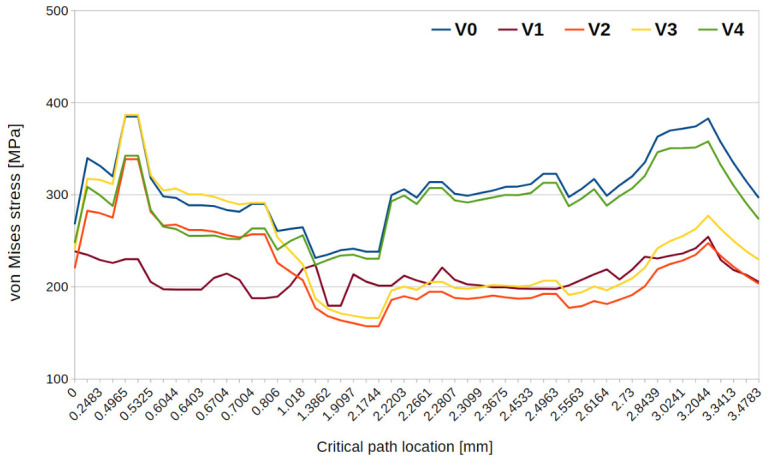
Distribution of von Mises stress values along the CP in all cases (V0–V4) of FEA model at 30-degree angle of attack.

**Figure 26:**
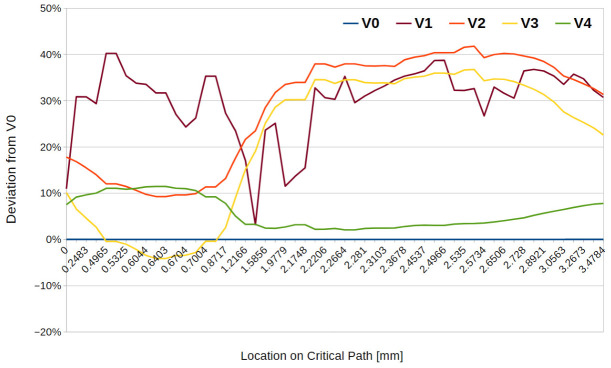
Distribution of relative differences of von Mises stress values compared to the base version (V0), the average deviation is 5.8% at V4 and 30.2%, 28.6%, 21.3% at V1, V2, V3, respectively.

Quantitative comparison between the model variants (Figure 21 and Figure 24) was performed by examining equivalent (von Mises) stress values along the critical path (CP) on the prosthesis structure under a 30° loading angle (Figure 25). The relative differences shown in Figure 26, again calculated with respect to the reference model (V0), indicated that the V4 configuration performed most consistently, exhibiting nearly equivalent stress responses with an average difference of 5.8%.

The comparative finite element analysis revealed that the oversimplified boundary conditions of V1 created an unrealistic mechanical environment with artificially amplified stress magnitudes, thereby demonstrating the importance of anatomically constrained conditions. The V2 and V3 configurations, characterised by greater global mandibular deformation, exhibited high stress accumulation at the rigid connection points of the bridge, where thin material bridges transmitted concentrated loads between elements. These findings highlight the risk of biomechanical misinterpretation when structural complexity is reduced excessively [3,4,22].

Nevertheless, the geometrically simplified V4 model—excluding the trabecular bone core—showed only minor deviations from the fully detailed reference configuration, with an average difference in stress magnitude of approximately 6%. This finding indicates that, under specific conditions, controlled model simplification can reduce computational demand without substantial loss of biomechanical fidelity [26,27]. Although different loading directions produced different local stress distributions, the present results suggest that, when simplification is applied appropriately, its influence on the overall finite element response remains limited.

### 3.2. Condensed Results

The PDL is essential for realistic displacement and force transfer; omitting it produces overly rigid and less physiological behaviour.

The retainer–pontic connector is the dominant stress-concentrating region and should be considered the main target of design optimisation.

Excessive simplification of geometry or boundary conditions leads to artificial stress amplification and distorted load paths.

The V4 model provides the best balance between computational efficiency and biomechanical fidelity, with only about 6% deviation from the full reference model.

The consistency of V4 model under both 0° and 30° loading suggests that this simplification strategy is robust for static loading conditions.

Critical Path-based analysis is more informative than single-point sampling because it captures stress gradients and regional behaviour.

## 4. Discussion

### 4.1. Reconstruction of the Anatomical Model

The present study demonstrates a high-fidelity, patient-specific workflow for reconstructing the mandible and dentition using NURBS-based parametric modelling derived from clinical CBCT data. The three-dimensional reconstruction pipeline—combining multiplanar CBCT segmentation (3D Slicer v5.6.2), orthogonal contour extraction, and NURBS surface lofting in PTC Creo 12.4—yielded a geometrically continuous, topologically consistent digital dentoalveolar complex. This approach addresses a longstanding limitation in dental biomechanics, namely the reliance on idealised or generic geometries that fail to capture interindividual anatomical variability [22,27]. By integrating orthogonal CT slices into a continuous NURBS surface network, our method preserves both external morphology and internal structural hierarchy—namely, the distinct cortical and trabecular bone compartments—while ensuring geometric smoothness and topological consistency. Excellent congruence with source slices across sagittal, axial, and coronal planes (Figure 5 and Figure 7) was evident in anatomical complex regions such as the mandibular canal, alveolar ridge, and root apices, where polygonal methods often perform less reliably [8,13,14]. This contrasts with conventional tessellated (STL-based) reconstructions [47], which frequently introduce stair-step artifacts and require excessive mesh refinement to approximate curvature, thereby compromising numerical efficiency and introducing discretisation errors [7,8,10,19].

The use of parametric curves fitted to segmented contours is consistent with established CAD standards in biomedical engineering [18] and enables systematic morphological modification for sensitivity analyses—a capability not readily available in conventional black-box segmentation tools. The close correspondence between reconstructed models and source CT slices (Figure 7) supports the anatomical fidelity of the present reconstruction pipeline. Notably, explicit representation of the lamina dura and alveolar bone architecture further enhances physiological relevance, a feature frequently omitted in prior finite element (FE) studies of tooth-supported prostheses [23,24]. In contrast to earlier FE investigations focused primarily on prosthesis loading with idealised geometries [22,27], the present method provides anatomically realistic tooth reconstruction, that may support more integrated analysis of mandibular biomechanics.

### 4.2. Clinical Model, Model Simplifications

A central contribution of this work lies in the systematic evaluation of model simplification strategies commonly employed in the FE literature. The present findings confirm that extreme simplifications—such as the monolithic rigid block (V1)—produce non-physiological stress states, with artificially elevated magnitudes and distorted load paths caused by the absence of periodontal compliance and bone heterogeneity [3,22]. Even models incorporating basic jaw morphology without internal trabeculation (V2) or with homogeneous cancellous bone (V3) exhibited exaggerated stress concentrations at crown–abutment interfaces, reflecting their inability to replicate natural load dissipation through the cancellous core. These results corroborate biomechanical principles articulated by Korioth and colleagues, who emphasised that the composite structure of bone critically modulates stress shielding and strain distribution [4,5].

Importantly, however, the V4 model—which retained the patient-specific mandibular geometry and PDL while omitting detailed trabecular architecture—yielded stress predictions within approximately 6% of the anatomically complete V0 reference model. This suggests that, for certain applications such as prosthesis-level stress screening, controlled simplification excluding fine trabecular detail may be justifiable, provided that cortical morphology, PDL representation, and tooth positioning are preserved. This finding offers a pragmatic compromise between computational tractability and biomechanical fidelity, and may support broader integration of FE analysis into digital treatment planning workflows.

### 4.3. Effect of Load Directions

To increase confidence in the observed deviation of approximately 6%, two finite element analyses were performed using different loading conditions. Application of static loads at 0° and 30° demonstrated that, although the absolute von Mises stress magnitudes and local stress distributions varied with loading angle—as expected under non-axial masticatory loading—the relative performance of the simplified models remained consistent.

The absolute von Mises stress distributions differed markedly between the two loading cases. Nevertheless, the relative deviation patterns were similar in both simulations. In particular, the V4 model maintained close agreement with the reference V0 model under both loading conditions, with mean deviations of 6.1% at 0° and 5.8% at 30°. These findings support the biomechanical suitability of the proposed geometric simplification, specifically the omission of detailed internal cancellous bone architecture under the present loading conditions.

This robustness suggests that, under the static loading conditions investigated here, preservation of the cortical envelope and periodontal ligament representation has a greater influence on load-transfer behaviour than detailed modelling of the trabecular microstructure. The localisation of peak von Mises stresses at the crown–bridge junctions in all model variants is also consistent with clinical observations identifying these regions as common fracture-prone sites in fixed partial dentures (FPDs) [6,30].

Importantly, the use of a distributed Critical Path sampling strategy, based on 55 points along a biomechanically relevant trajectory, provided a more informative assessment than conventional single-node reporting. This approach captured not only peak stress values but also stress gradients, which may be relevant to fatigue-related failure mechanisms [25]. The similar deviation profiles obtained under both loading conditions further suggest that simplification protocols that perform consistently under vertical loading may also remain stable under moderate off-axis loading. However, dynamic, cyclic, and patient-specific functional loading scenarios should be investigated separately before broader clinical generalisation is considered.

### 4.4. Effect of PDL Characteristics on Biomechanical Outcomes

Although many finite element studies do not explicitly incorporate the periodontal ligament (PDL), it is now widely recognised as a key structure governing the transmission of occlusal forces from the teeth to the alveolar bone. The PDL plays an essential role in reproducing physiological tooth mobility and realistic load distribution in finite element analysis (FEA) of dento-alveolar structures [33,38]. Nevertheless, despite the increasing number of studies that include the PDL in computational models, its influence on predicted biomechanical behaviour remains incompletely understood, largely because of differences in geometric representation, material assumptions, loading conditions, and boundary definitions.

Various analytical approaches and modelling assumptions have been proposed to describe the complex biomechanical response of the PDL [48]. Earlier studies predominantly employed homogeneous material formulations, including linear or nonlinear, isotropic or anisotropic, and elastic or poroelastic models [33,39]. Although these approaches provided an important methodological basis for dental FEA, they often simplified the intrinsically nonlinear, viscoelastic, and fluid-permeated nature of the ligament. More recent studies have attempted to improve physiological realism by introducing hyperelastic formulations, including models that exhibit nearly linear displacement behaviour under tensile loading and hydrostatic pressure-dependent behaviour under compression [34,40,41,42,43], as well as Ogden-type hyperelastic models capable of representing large-strain nonlinearities [35]. At the same time, comparative FEA studies have indicated that carefully calibrated linear elastic material models may still provide clinically meaningful stress and strain predictions when combined with anatomically realistic geometry and appropriate boundary conditions [49].

In the present study, the PDL geometry was reconstructed using a precise thin-offset algorithm applied to the root surfaces, resulting in an average thickness of 0.2 mm. This value is consistent with physiological measurements reported in the literature, where PDL thickness is typically described within the range of approximately 0.15–0.38 mm [31,33,35]. Although the PDL demonstrates time-dependent viscoelasticity under orthodontic loading [40,41,42,43] and pronounced nonlinear behaviour under physiological loading [33], the sensitivity analysis and comparative hyperelastic simulations performed in this study (Figure 12) support the adequacy of a linear elastic approximation for static occlusal loading scenarios. This conclusion is consistent with previous quasi-static FEA studies [33,37].

Accordingly, the PDL was represented as a homogeneous, quasi-incompressible, linear elastic layer with an elastic modulus of 67 MPa, a Poisson’s ratio of 0.49, and a thickness of 0.2 mm. This modelling strategy provides a pragmatic balance between biomechanical relevance and computational efficiency, particularly in patient-specific simulations where geometric complexity, mesh quality, and solution time are critical constraints [3,5,31,34]. The wide range of elastic modulus values reported for the PDL in the literature, approximately 0.15–175 MPa [37,44,45], reflects substantial methodological variability in experimental measurement and numerical calibration. The value selected in the present study lies within the mid-to-high range reported for masticatory-force conditions and demonstrated stable convergence across a clinically relevant modulus interval of 50–175 MPa, with deviations remaining within approximately ±1.9%.

The incorporation of the PDL layer proved to be biomechanically crucial: the V1b configuration showed approximately twice the prosthesis displacement observed in the rigidly fixed V1a model. This finding confirms that even a simplified linear elastic representation of the PDL can substantially improve the physiological plausibility of the predicted displacement and load-transfer behaviour under static loading conditions. The comparative conclusions regarding model variations (V1–V4) and geometric simplification strategies remain robust within reasonable ranges of PDL material properties. Together with findings reported in the literature, these results suggest that although absolute stress values are sensitive to the selected PDL modulus, the overall comparative trends remain consistent. Further studies may help clarify the distinction between global agreement (e.g., overall displacement patterns) and potential local effects, particularly with regard to stress sensitivity in the tooth–PDL interface region. Because the present modelling approach does not account for viscoelastic, nonlinear, bilinear, or fatigue-related behaviour, future studies should incorporate dynamic cyclic loading conditions, advanced multiphysics models—including fluid–structure interaction within the PDL—as well as experimental validation and investigations in larger patient populations to enhance the translational robustness and clinical relevance of the approach.

### 4.5. Model Validation and Limitations

Direct experimental validation of internal stress fields within the human jaw remains technically challenging. Consequently, the validity of finite element (FE) models in this context relies largely on indirect forms of verification. In the present study, three levels of verification were applied: (1) geometric fidelity, demonstrated by slice-by-slice CT–CAD alignment (Figure 7); (2) methodological consistency with published literature values for jaw displacement and stress magnitudes under a 500 N load [31,32,33,34,35]; and (3) internal numerical consistency through adaptive mesh convergence.

The peak deformation observed in the baseline model (V0), 0.74 mm, falls within the physiological range of mandibular flexion reported in strain-gauge and photoelastic studies [31,32]. In addition, localisation of stress maxima at the crown–abutment interfaces are consistent with known clinical failure patterns [6] and with previous computational findings [30]. Although in vivo strain measurements would further strengthen the validation framework, the agreement with established biomechanical principles and published comparative data supports the usefulness of the model for relative analyses.

The von Mises stresses and distributions under the 500 N loading condition (peak ~507 MPa) are consistent with previous finite element analysis (FEA) studies on tooth-retained prostheses [31,32]. Secondary FE analysis according to ISO 14801:2016 [46] experimentally confirmed the reliability of the simplification approach. The FE model was specifically developed for the comparative evaluation of variations in material assignment. The applied geometric boundary conditions, such as the fixed displacement constraint at the superior aspect of the mandible, reduced computational time but also led to slightly higher maximum von Mises stress values and limited displacement. Simplifications of these conditions, including the omission of kinematic constraints such as elastic connections and surface friction, restricted the model’s ability to simulate the mandibular environment in detail; however, the approach remained suitable for comparative analysis. The tooth–PDL interaction played only a marginal role in the interpretation of the global results. This limitation is acceptable only within the scope of the present study and should be addressed through further refinement and investigation in future work. Another limitation of the study is the simplified material distribution, as the FE model assumed homogeneous volumes for different material regions, whereas real bone exhibits continuous transitions between them.

Although the numerical results are consistent with literature values (e.g., von Mises peaks of ~507 MPa [31,32]), limitations include the lack of direct in vitro or in vivo validation (e.g., strain gauge measurements or micro-CT-based fatigue studies). Reliance on a single subject cone beam computed tomography (CBCT) scan introduces between-patient variability bias and limits generalisability; multicenter, multi-patient datasets are needed to account for anatomical diversity, bone quality, and restorative variations. The 500 N static load at an asymmetric angle approximates peak masticatory forces but omits multidirectional dynamics, masticatory cycles, and muscle vectors.

The single-case design, focusing on one patient’s mandibular morphology and a specific edentulous premolar–molar bridge configuration, precludes population-level inferences. Consequently, the findings are primarily applicable to similar FPD scenarios in otherwise healthy mandibles. Extrapolation to periodontally compromised cases, extended multi-unit prostheses, or diverse demographic groups (e.g., elderly patients, reduced bone quality) should be undertaken with caution. Moreover, the analysis addresses immediate stress distribution under static loads, without incorporating fatigue, creep, bone remodelling, or probabilistic failure assessment (e.g., Weibull analysis, stochastic material tolerances).

The NURBS–FEA framework presented in this study may offer valuable support for the development of patient-specific biomechanical modelling systems. Furthermore, the use of validated geometric simplifications may contribute to the optimisation of future digital biomechanical design workflows. Nevertheless, FEA should currently be viewed primarily as a hypothesis-generating and decision-support methodology, rather than as a direct and definitive predictor of clinical outcomes.

### 4.6. Effects of Geometric Simplification on Results

Comparative FE analysis demonstrates that controlled geometric modification profoundly influences the predicted mechanical response of a three-unit fixed partial denture (FPD) system [3,34]. Systematic evaluation of the baseline anatomical configuration (V0) against simplified variants (V1–V4) revealed that geometric detail and boundary-condition realism are primary determinants of the stress–strain state [3,31]. The fully detailed V0 model, incorporating a physiologically realistic mandible, teeth, and periodontal ligament (PDL), exhibited a maximum von Mises stress of 507 MPa and peak deformation of 0.74 mm along the mandibular midsagittal plane. Stress concentrations predominated at the crown–bridge interfaces, underscoring their role as critical sites where stiffness discontinuities and geometric transitions amplify local stresses [1,2,3,27]. These findings are consistent with clinical observations and earlier simulations identifying connector zones as prone to fatigue and failure [30,33].

In contrast, the oversimplified V1 model—with idealised boundary conditions—produced artificially elevated stresses and unrealistic deformation patterns. The rigid cortical structure and omission of anatomically relevant features constrained mandibular mobility, and failed to reproduce functional behaviour under masticatory loading [3,31,34]. This illustrates how excessive simplification of support conditions can lead to overestimation of peak stresses and, consequently, to misleading assessments of prosthesis safety and longevity [3,27].

Intermediate variants V2 and V3 allowed greater mandibular deformation but exhibited pronounced stress accumulation at rigid connector regions, particularly thin geometric connections transmitting inter-element loads. These stress peaks arose from non-physiological load pathways induced by simplified geometries and connectivity, highlighting risks of misinterpretation when structural continuity, thicknesses distribution, and material assignment are not represented with sufficient fidelity [3,31,34].

Notably, the V4 variant, which omitted the trabecular bone core while preserving cortical structures and overall anatomy, showed minimal deviation from V0, with an average stress difference of approximately 6% average stress differences at points A–D. This finding is consistent with previous reports suggesting that, under certain loading conditions, the cortical shell governs segmental stiffness sufficiently to permit omission of trabecular detail without substantial error [21,26]. From a practical perspective, such model reduction strategies may reduce computational demand when they are biomechanically justified and benchmarked against more detailed reference models [3,27,31,34]. Whereas coarse idealisations such as V1–V3 distort the predicted mechanical response, targeted simplification as implemented in V4 appears to offer a useful balance between efficiency and realism, particularly for engineering workflows and larger cohort studies in which fully detailed reconstructions may be impractical.

It is important to emphasise that the pilot measurements were derived from a simplified biomechanical simulation that assumed homogeneous material behaviour and static loading conditions, without accounting for time-dependent physiological processes. While these simplifications reduce the physiological complexity of the model, they facilitate the comparative analysis of a large number of samples. This approach may serve as a foundation for further experimental validation of the method by means of dynamic fatigue testing in future studies.

### 4.7. Clinical Significance, Clinical Translation, and Generalisability

The clinical relevance of this study lies in the development of a robust, patient-specific digital framework that combines high-accuracy anatomical reconstruction with biomechanical simulation. This approach may provide information of practical value in restorative dentistry by supporting improved understanding of stress distribution in fixed partial dentures (FPDs) and by helping to identify design-related factors associated with complications such as abutment overload or connector failure [31,32,49].

The results of this pilot study indicate that neglecting the heterogeneity of the periodontal ligament (PDL) or mandibular structure may lead to substantial overestimation of stress, potentially resulting in unnecessary design modifications or excessive material use. In addition, the performance of the V4 model suggests that clinicians and dental technicians may, in the future, be able to use simplified yet sufficiently detailed finite element workflows for digital prosthetic design. Such an approach could allow computational resources to be concentrated on critical stress regions, such as connector zones, rather than on exhaustive modelling of internal bone detail.

Identification of the retainer–pontic connector as a persistent stress-concentrating region also suggests a clear direction for design optimisation. Potential strategies may include increasing connector cross-sectional area and using high-strength materials in these regions [6,30].

More broadly, by aligning simulation studies more closely with patient-specific anatomical conditions, this approach moves beyond assumptions based solely on population averages. In principle, this may support restoration design tailored to individual anatomical and biological characteristics, such as bone quality or periodontal status, and may contribute to more refined personalised risk assessment.

Beyond immediate case-specific planning, the NURBS–FEA pipeline may open broader opportunities in digital dentistry. Its parametric adaptability facilitates rapid prototyping of prosthetic variants and systematic exploration of design alternatives, including retainer–pontic connector geometry, material combinations, and abutment configurations, under controlled biomechanical conditions. Integration with AI-driven optimisation algorithms may further support automated material selection and geometric refinement, while incorporation into CBCT-based clinical workflows may streamline data acquisition and model generation [6,11,15,29]. Future development could include dynamic loading simulations, multiphysics modelling of PDL fluid dynamics, and validation in larger patient cohorts to support more reliable patient-specific treatment planning. Collectively, these directions point toward a broader transition from empirically guided to computation-assisted prosthodontics, in which individualised biomechanical simulations may increasingly inform clinical decision-making and long-term rehabilitation strategies.

## 5. Conclusions

A patient-specific NURBS–FEA workflow was developed for anatomically realistic reconstruction and biomechanical evaluation of mandibular fixed partial dentures. The findings show that preserving cortical anatomy and the PDL is critical for accurate stress prediction, whereas omission of detailed trabecular architecture of the bone may be acceptable for simplified clinical simulations. The PDL proved essential for physiological load transfer, and a linear elastic representation was adequate under static occlusal loading. Stress concentrations consistently occurred in the retainer–pontic connector region, highlighting this area as a primary target for design optimisation. In the future, these results may provide a methodological basis for biomechanical simulation-based and clinically applicable digital prosthesis design. However, further validation is required in larger cohorts and under dynamic loading conditions. The experimental validation of the current finite element model is not yet considered to be adequately supported; therefore, the model results cannot be directly interpreted as a predictive description of clinical biomechanical behaviour.

## Data Availability

The CBCT data analysed in this study were obtained from a single volunteer (own study, no IRB required) and are not publicly available due to privacy restrictions. The derived NURBS models (STL/IGES formats), finite element analysis input files (Creo Simulate scripts), and processed datasets (stress/strain outputs), model parameters, and reconstruction workflows are available upon request from the corresponding author [piros.attila@nje.hu].
